# Sympatric non-biting flies serve as potential vectors of zoonotic protozoan parasites on pig farms in China

**DOI:** 10.1186/s13071-025-06686-2

**Published:** 2025-02-18

**Authors:** Yufeng Liu, Pitambar Dhakal, Wenyan Hou, Fa Shan, Nanhao Wang, Bin Yang, Huikai Qin, Xiaoying Li, Rongjun Wang, Longxian Zhang, Sumei Zhang, Junqiang Li

**Affiliations:** 1 College of Veterinary Medicine, Henan Agricultural University, Zhengzhou, 450046 Henan China; 2 International Joint Research Laboratory for Zoonotic Diseases of Henan, Zhengzhou, 450046 China; 3 Key Laboratory of Quality and Safety Control of Poultry Products, Ministry of Agriculture and Rural Affairs, Zhengzhou, 450046 China; 4 Rural Industry Development Center of Shangqiu City, Shangqiu, 476100 China

**Keywords:** Non-biting flies, Pig farms, *Cryptosporidium* spp., *Enterocytozoon bieneusi*, *Blastocystis sp*.

## Abstract

**Background:**

*Cryptosporidium* spp., *Enterocytozoon bieneusi*, and *Blastocystis* sp. are common enteric parasites in humans and pigs. Ascertaining whether non-biting flies (NBFs) serve as potential vectors of these parasites on pig farms is a crucial aspect of disease control.

**Methods:**

Non-biting flies were collected and identified by morphology analysis together with sequence analysis of the mitochondrial cytochrome* c* oxidase I (CO1) gene as confirmation. In a cross-sectional study, the small-subunit ribosomal RNA (*SSU* rRNA) gene of *Cryptosporidium* spp., the internal transcribed spacer region (ITS) region of *E. bieneusi* and the* SSU* rRNA gene of *Blastocystis* sp. were investigated in fresh pig fecal samples and sympatric NBFs.

**Results:**

The results revealed the occurrence of five species of NBFs (*Musca domestica*, 91.2%; *Lucilia sericata*, 5.8%; *Chrysomya megacephala*, 1.7%; *Aldrichina grahami*, 0.6%; *Helicophagella melanura*, 0.6%) in the collected pig fecal samples. The prevalence of *Cryptosporidium* spp., *E. bieneusi* and *Blastocystis* sp. on the body surface of NBFs was 0.6% (2/342), 4.4% (15/342) and 20.8% (71/342), respectively. Similarly, the prevalence of these parasites in the lysates of NBFs (= in vivo carriage) was 0% (0/342), 2.7% (9/342) and 10.5% (36/342), respectively. The prevalence of *Cryptosporidium* spp., *E. bieneusi* and *Blastocystis* sp. in pigs from which fly samples were collected was 2.3% (41/1794), 12.6% (226/1794) and 30.8% (553/1794), respectively. The zoonotic *Cryposporidium suis*/*C. scrofarum*, *E. bieneusi* ITS genotypes EbpA/EbpC and *Blastocystis* sp. subtypes ST1/ST3/ST5 were identified in both NBFs and pig feces. NBFs were found to carry *E. bieneusi* and *Blastocystis* sp. on their body surface as well as in the lysates.

**Conclusions:**

These findings demonstrate the role of NBFs as potential vectors in the dissemination of these zoonotic parasites in pig farms, and also highlight the possibility of their transmission to humans.

**Graphical Abstract:**

**Figure:**
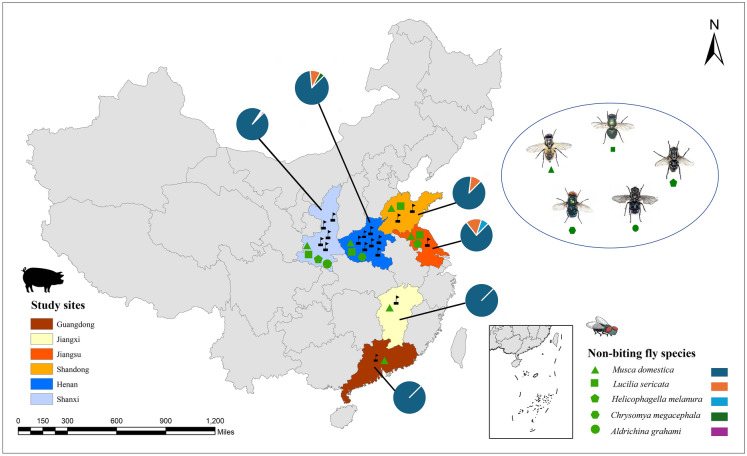

**Supplementary Information:**

The online version contains supplementary material available at 10.1186/s13071-025-06686-2.

## Background

Non-biting flies (NBFs) generally feed and breed in the field and are frequently found in areas where human and animal activities are common, including livestock and poultry farms [1]. Parasites can adhere to the body hairs, wings and appendages of NBFs, or even be ingested by these flies, and, therefore, NBFs can serve as vectors [2, 3]. NBFs are considered to be the carriers of hundreds of pathogens, including viruses, bacteria, fungi and parasites of both veterinary and medical importance [1, 3].

*Cryptosporidium* spp*.*, *Enterocytozoon bieneusi* and *Blastocystis* sp*.* are important and common zoonotic protozoan parasites in humans, pigs and other animals [4]. They are transmitted mainly via the fecal–oral route (contaminated water or food), causing acute gastroenteritis, abdominal pain and diarrhea in infected hosts, particularly in immunocompromised hosts [5]. To date, more than 40 species and more than 120 genotypes of *Cryptosporidium* have been identified on the basis of the small subunit ribosomal RNA gene (*SSU* rRNA) and glycoprotein (*gp*60) genes [6, 7], of which *Cryptosporidium suis* and *C. scrofarum* are the most common* Cryptosporidium* species identified in pigs and reported in humans [8]. More than 500 *E. bieneusi* genotypes have been reported based on identification using the internal transcribed spacer (ITS) locus, of which more than 60 different genotypes have been identified in pigs, with the majority of them also found in humans [9–11]. Similarly, 32 subtypes of *Blastocystis* sp*.* have been identified on the basis of *SSU* rRNA, of which nine (ST1–ST8 and ST12) are zoonotic subtypes [12–14].

Swine feces and associated waste are the preferred feeding and breeding sites for NBFs, and many types of NBFs have been documented on and around livestock farms [1]. The transmission of NBFs is remarkable, as they carry many zoonotic protozoan parasites in the environment [15–17]. Therefore, we investigated the diversity of the sympatric NBFs in pig farms and their potential role in the transmission of zoonotic *Cryptosporidium* spp*.*, *E. bieneusi* and *Blastocystis* sp. This study provides insight into the potential role of NBFs as carriers (vectors) of protozoan pathogens, and the importance of their control in pig farms as a means to strengthen public health safety.

## Methods

### Sample collection

**Fig. 1:**
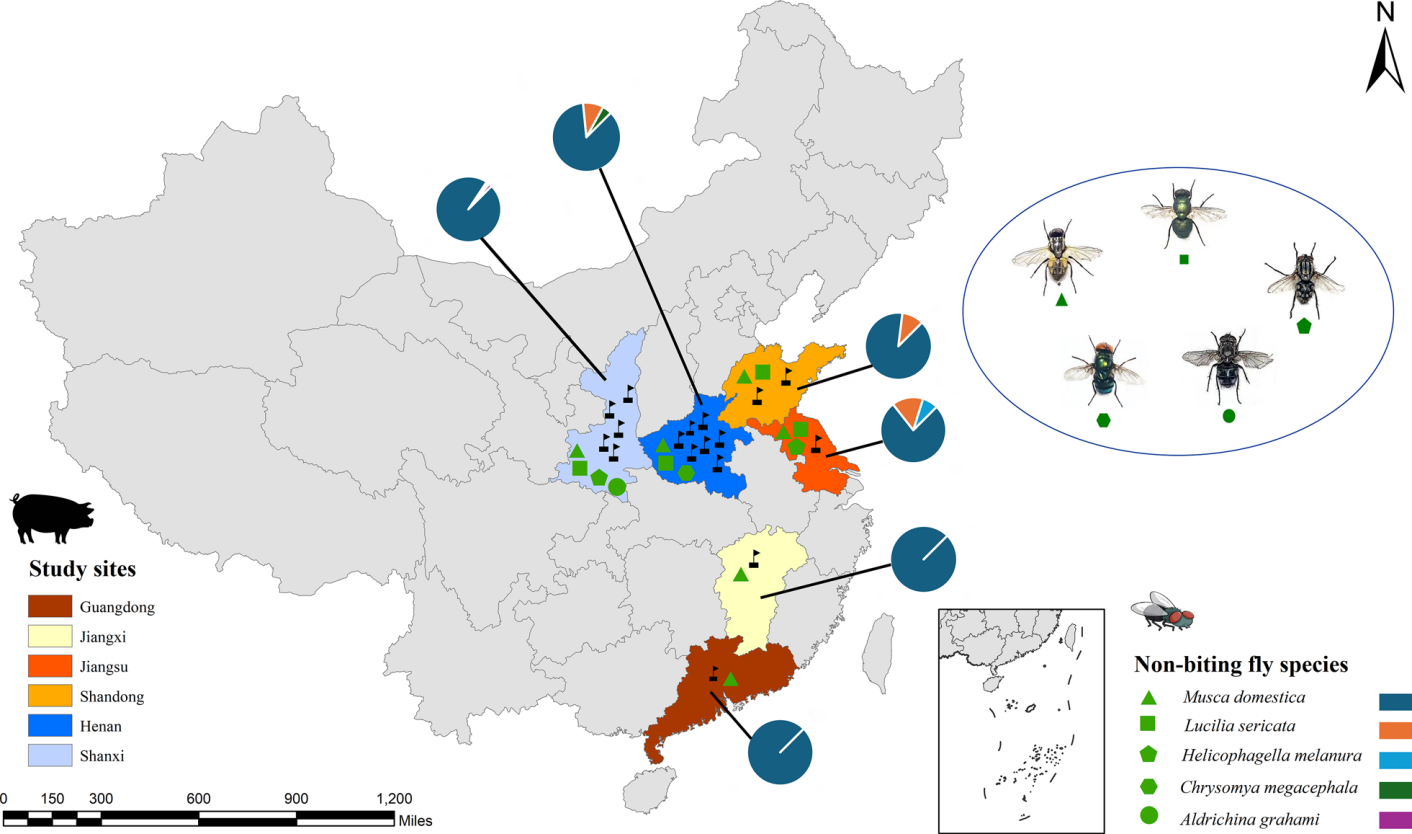
Location map of the study area. Six provinces from where the pig fecal samples and the sympatric non-biting flies (NBFs) were collected are depicted with the respective legends. The pie chart indicates the species-specific proportion of collected HBFs in the six provinces. The study area map was created using ArcGIS Pro 3.1.6

In this cross-sectional study conducted from May 2021 to November 2022, a total of 3420 NBFs were captured from 10 small-scale farms (< 100 pigs) and seven large-scale farms (> 100 pigs) situated in six provinces of China (Fig. 1), using fly trap paper and/or fly-fishing nets. The fly traps were placed on the floor of the farm building (2 m away from the areas where pigs were held) and on the inner and outer surface of the farm walls (1 m above from the ground), at locations where pigs could not disturb them. Traps were also set close to the windows and doors, especially near manure and also hung on the ceiling where flies generally rest. Likewise, fly-trap nets were swept in the air in and around the farm to collect flying NBFs. The captured NBFs were picked out of the traps and directly transferred into the zip lock bags in dry condition. At the same time, 1794 fresh fecal samples from pigs were collected from the same pig farms (Additional file 1: Table S1). To avoid cross contamination, only freshly passed fecal samples were collected via rectal sampling or immediately after defecation. All collected samples were kept in a refrigerated ice foam box, immediately transported to the International Joint Research Laboratory for Zoonotic Diseases of Henan within 48 h and then stored in a freezer at 4 °C.

### Identification of sympatric NBF species

Each sympatric NBF was examined under a stereo microscope and classified to the species level on the basis of morpho-taxonomical characteristics such as body size, body color and thoracic and abdominal structures [2, 18]. The identity of the NBF species was confirmed by a molecular method, as follows. The flies were pooled into 342 pools, each containing 10 flies of the same species on the basis of a similar morphology. Each pool of NBFs was crushed in a tissue grinder, and DNA was extracted from the tissue using the TlANamp Genomic DNA Kit (China) following the manufacturer’s instructions. The mitochondrial cytochrome* c* oxidase I (COl) gene of NBFs was amplified using primers reported in a previous study [19] (Additional file: Table S2).

### Isolation of parasites from the body surface of the NBFs

All collected NBFs were divided into 342 sample pools, where one pool was defined as a collection of 10 flies with similar morphology and therefore considered to be a single sample. An appropriate amount of physiological saline (0.9% NaCl) was added to each pool, and the mixture was then placed in a shaker for 5 min without damaging the NBFs body [20, 21]. All eluants (body washes) were transferred to new tubes, followed by centrifugation. The supernatant was discarded, and the precipitate was used separately for parasite identification. This procedure was repeated 3 times to ensure the recovery of parasites attached to the body surface of the NBFs.

### Isolation of parasites in the lysate of NBFs

After the body surface of each pool of flies had been washed to isolate the parasites from their external body surface, each pool of flies was transferred into a new tube. Then, each pool of NBFs was crushed with a tissue mortar so that the parasites they had ingested into the gut could be released into the respective lysates (homogenates) [20]. In the present study, the term ‘lysate’ mainly refers to the gut contents of the NBFs which contain the parasites the NBFs have ingested. The lysates were then immediately filtered through a 198-μm sieve, and the filtrates were subsequently centrifuged at 1500 *g* for 5 min at room temperature [20]. The supernatant was discarded after centrifugation, and the precipitates were retained for parasite identification [2, 22]. This procedure was repeated 3 times for each pool to ensure the recovery of parasites from the NBFs in lysates.

### DNA extraction and PCR amplification of parasites from NBFs

Genomic DNA from the body surface washings of the NBFs and in lysate eluants was extracted using the DNeasy® PowerSoil® ProKit following the manufacturer’s instructions (QIAGEN, Hilden, Germnay), and the extracted DNA from each pool was stored at – 20 °C. The PCR amplification of extracted DNA was carried out using species-specific primers targeting the 18S rRNA gene of *Cryptosporidium* spp., the ITS region of *E. bieneusi* and the *SSU* rRNA gene of *Blastocystis* sp., as described previously [23–25] (Additional file 2: Table S2). The PCR products were electrophoresed in a 1.5% agarose gel to identify the positive amplicons.

### DNA extraction and PCR amplification of parasites from pig fecal samples

The genomic DNA of the pathogens was extracted from freshly collected pig fecal samples (20–200 mg) using E.Z.N.A. Stool DNA Kits (Omega Biotek Inc., Norcross, GA, USA). The PCR amplification conditions and identification process were the same as those described above for parasites extracted from NBF samples.

### DNA sequence analysis

All positive PCR amplicons were submitted to a commercial sequencing company (SinoGenoMax Biotechnology Co., Ltd., Beijing, China) for bidirectional sequencing on an ABI-PRISM™ 3730XL DNA Analyzer using the BigDye Terminator v3.1 Cycle Sequencing Kit (Applied Biosystems, Thermo Fisher Scientific, Waltham, MA, USA). The alignments were performed using MEGA 7.0 (https://megasoftware.net), followed by a Basic Local Alignment Search Tool (BLAST) search of the homology sequence in the GenBank database to determine the genotype and subtype of the parasites.

### Statistical analysis

Data were statistically analyzed using IBM SPSS Statistics version 22 software (SPSS IBM, Armonk, NY, USA), and the Chi-square (χ^2^) test was used to analyze the prevalence of NBF species and the intestinal protozoan parasites carried by NBFs in different sampling regions. In addition, the prevalence of identified parasites in pigs was compared on the basis of the host’s age groups and sampling sites. Statistical significance was set at* P* < 0.05.

## Results

### Characteristics of the study population of pigs and NBFs

A total of 3420 NBFs were collected from the sampled pig farms, among which 1020 (29.8%) were from large-scale farms and 2400 (70.2%) were from small-scale farms (Fig. 1). Similarly, of the 1794 pig fecal samples collected in total, samples from pigs at various developmental stages, such as suckling piglets (< 21 days;* n* = 248, 13.8%), weaning pigs (1–2 months;* n* = 444, 24.7%), finishing pigs (3–6 months;* n* = 670, 37.3%) and sows (> 6 months;* n* = 432, 24.1%), were included. Regarding pig farm types, 53.6% (*n* = 961) of the fecal samples were collected from small-scale farms, and 46.4% (*n* = 833) were collected from large-scale farms. The overall sociodemographic characteristics of the NBFs and pigs included in this study are shown in Table 1.

**Table 1:** Overall sociodemographic characteristics of the non-biting flies and pigs included in the study

Non-biting flies (NBFs)				Pigs			
Factors	No. of collected flies	No. of fly batches	Proportion	Factors	No. of pig farms	No. of pig fecal samples	Proportion
*Location*				*Location*			
Henan	1350	135	39.5%	Henan	7	460	25.6%
Shaanxi	1270	127	37.1%	Shaanxi	5	942	52.5%
Shandong	380	38	11.1%	Shangdong	2	69	3.8%
Guangdong	60	6	1.8%	Guangdong	1	108	6.0%
Jiangsu	130	13	3.8%	Jiangsu	1	108	6.0%
Jiangxi	230	23	6.7%	Jiangxi	1	107	6.0%
*Fly species*				*Pig development stage*			
*Musca domestica*	3120	312	91.2%	Suckling piglets (< 21 days)	–	248	13.8%
*Lucilia sericata*	200	20	5.8%	Weaning pigs (1–2 months)	–	444	24.7%
*Chrysomya megacephala*	60	6	1.7%	Finishing pigs (3–6 months)	–	670	37.3%
* Aldrichina grahami*	20	2	0.6%	Sows (> 6 months)	–	432	24.1%
* Helicophagella melanura*	20	2	0.6%				
*Breeding scale*				*Breeding scale*			
Large scale	1020	102	29.8%	Large scale	7	833	46.4%
Small scale	2400	240	70.2%	Small scale	**10**	961	53.6%
Total	3420	342	100%	Total	17	1794	100%

### Identification of NBF species

On the basis of morphological features and molecular methods, five NBF species (*Musca domestica*, *Aldrichina grahami*, *Chrysomya megacephala*, *Helicophagella melanura* and *Lucilia sericata*) were identified. Among the total NBFs collected, *M. domestica* was collected from farms in all the provinces and comprised the highest proportion of NBFs (91.2%; 3120/3420), followed by *L. sericata* (5.8%;* n* = 200), *C. megacephala* (1.8%;* n* = 60) and equal proportions (0.6%) of *A. grahami* (*n* = 20) and *H. melanura* (*n* = 20) (Table 1).

### Prevalence and genotypes of parasites on the body surface of NBFs

Among the 342 pools of NBFs, the prevalence rates of *Cryptosporidium* spp., *E. bieneusi* and *Blastocystis* sp. on the body surface of NBFs were 0.6% (2/342), 4.4% (15/342) and 20.8% (71/342), respectively (Table 2). *Cryptosporidium* spp. (0.6%, 2/342) identified on the body surface of NBFs were confirmed to be *C. suis* (*n* = 1) and *C. scrofarum* (*n* = 1). *Cryptosporidium suis* was identified on the body surface of *Musca domestica* collected from large-scale pig farms in Henan Province, whereas *Cryptosporidium scrofarum* was identified on the body surface of *H. melanura* captured from large-scale pig farms in Jiangsu Province. Regarding *E. bieneusi* (1.7%, 15/342), only the EbpC (*n* = 13) and EbpA (*n* = 2) genotypes were identified, with EbpC the dominant genotype. Similarly, among the *Blastocystis* sp. (20.8%, 71/342), only the ST3 (*n* = 56) and ST5 (*n* = 15) genotypes were identified, with ST3 the dominant genotype.

**Table 2:** Prevalence and genotypes of intestinal parasites on the body surface of non-biting flies)

Factors	Number of collected flies	Number of fly batches	Total number of positives	*Cryptosporidium * spp.		*Enterocytozoon bieneusi*		*Blastocystis* sp.	
				Number of positives	*Cryptosporidium* spp. (*n*)	Number of positives	Genotypes (*n*)	Number of positives	Subtypes (*n*)
*Locations*									
Henan	1350	135	37	1	*C. suis* (1)	1	EbpC (1)	35	ST3 (24), ST5 (11
Shaanxi	1270	127	14	–	–	8	EbpC (8)	6	ST3 (6)
Shandong	380	38	9	–	–	2	EbpC (2)	19	ST3 (18), ST5 (1)
Guangdong	60	6	5	–		–	–	5	ST3 (4), ST5 (1)
Jiangsu	130	13	10	1	*C. scrofarum* (1)	4	EbpC (2), EbpA (2)	5	ST3( 4), ST5 (1)
Jiangxi	230	23	1	-	-	–	–	1	ST5 (1)
*Fly species*									
*Musca domestica*	3120	312	72	1	*C. suis* (1)	15	EbpC (13), EbpA (2)	56	ST3 (43), ST5 (13)
*Lucilia sericata*	200	20	15	–	–	–	–	15	ST3 (13), ST5 (2)
*Chrysomya megacephala*	60	6	–	–	–	–	–		
*Aldrichina grahami*	20	2	–	–	–	–	–	–	–
*Helicophagella melanura*	20	2	1	1	*C. scrofarum* (1)	–	–	–	–
*Breeding scale*									
Large scale	1020	102		2	*C. suis* (1), *C. scrofarum* (1)	6	Ebp C (4), EbpA (2)	20	ST3 (16), ST5 (4)
Small scale	2400	240		–	–	9	EbpC (9)	51	ST3 (40), ST5 (11)
Total	3420	342		2	*C. suis* (1), *C. scrofarum* (1)	15	EbpC (13), EbpA(2)	71	ST3 (56), ST5 (15)

### Prevalence and genotypes of parasites in lysates of NBFs

The in vivo identified parasites in NBF lysates were *E. bieneusi* and *Blastocystis* sp., with respective prevalence rates of 2.7% (9/342) and 10.5% (36/342), whereas *Cryptosporidium* spp. were not detected (Table 3) in the NBF lysates. With respect to *E. bieneusi* (*n* = 9), only two genotypes, EbpC (*n* = 8) and EbpA (*n* = 1), were identified. Among the total number of detected *Blastocystis* sp. (*n* = 36), three genotypes, namely, ST3 (*n* = 20), ST5 (*n* = 14) and ST1 (*n* = 2), were identified.

**Table 3:** Prevalence and genotypes of intestinal parasites in the lysates of non-biting flies

Factors	Number of collected flies	Number of fly batches	Total number of positives	*Cryptosporidium *spp.		*Enterocytozoon bieneusi*		*Blastocystis* sp.	
				Number of positives	Species	Number of positives	Genotypes (*n*)	Number of positives	Subtypes (*n*)
*Location*									
Henan	1350	135	20	–	–	5	EbpC (4), EbpA (1)	15	ST3 (12), ST5 (3)
Shaanxi	1270	127	7	–	–	2	EbpC (2)	5	ST1 (2), ST3 (2), ST5 (1)
Shandong	380	38	10	–	–	2	EbpC (2)	8	ST3 (2), ST5 (6)
Guangdong	60	6	5	–	–	–	–	5	ST3 (3), ST5 (2)
Jiangsu	130	13	1	–	–	–	–	1	ST5 (1)
Jiangxi	230	23	2	–	–	–	–	2	ST3 (1), ST5 (1)
*Fly species*									
* Musca domestica*	3120	312		–	–	9	EbpC (8), EbpA (1)	26	ST3 (15), ST5 (9), ST1 (2)
* Lucilia sericata*	200	20	6	–	–	–	–	6	ST5(3), ST3(3)
* Chrysomya megacephala*	60	6	0/1	–	–	–	–	3	ST3(2), ST5(1)
* Aldrichina grahami*	20	2	1	–	–	–	–	1	ST5(1)
* Helicophagella melanura*	20	2	–	–	–	–	–	–	–
*Breeding scale*									
Large scale	1020	102	14	–	–	4	EbpC(3), EbpA (1)	10	ST3(6), ST5(4)
Small scale	2400	240	31	–	–	5	EbpC (5)	26	ST3 (14), ST5 (10), ST1 (2)
Total	3420	342	45	–	–	9	EbpC (8), EbpA(1)	36	ST3 (20), ST5 (14), ST1 (2)

### Infection prevalence and genotypes of intestinal protozoan parasites in pigs

Our investigation on the co-occurrence of the three protozoan parasites in 1794 pig fecal samples showed that the infection rates of *Cryptosporidium* spp*.*, *E. bieneusi* and *Blastocystis* sp*.* were 2.3% (41/1794), 12.6% (226/1794) and 30.8% (553/1794), respectively (Table 4). A total of 41 (2.3%, 41/1794) fecal samples tested positive for the 18S rRNA gene of *Cryptosporidium* spp*.*, with *C. suis* and *C. scrofarum* identified in 21 and 20 of these fecal samples, respectively; the results confirmed by DNA sequencing. A total of 226 fecal samples (12.6%, 226/1794) tested positive for the ITS sequence of *E. bieneusi*. The genotypes identified were EbpC (*n* = 141), EbpA (*n* = 51), PigEBITS5 (*n* = 12), CHG19 (*n* = 5), HIJIV (*n* = 4), Henan-IV (*n* = 4), CHS5 (*n* = 4), O (*n* = 3), BEB6 (*n* = 1) and H (*n* = 1), among which EbpC was the dominant genotype. Similarly, 553 fecal samples (30.8%, 553/1794) tested positive for the *SSU* rRNA gene of *Blastocystis* sp*.*, with three gene subtypes, namely ST5 (*n* = 508), ST3 (*n* = 24) and ST1 (*n* = 21) confirmed; the dominant subtype was ST5 (Table 4). Statistical analysis revealed highly significant differences in the prevalence of these three intestinal protozoan infections in pigs with respect to both different regions and different age groups of pigs (*P* < 0.01) (Table 4). A comparative analysis of these parasites in relation to the developmental stages of pigs revealed that all developmental categories were infected with *C. scrofarum*, whereas weaning, finishing and sows were infected with both *C. scrofarum* and *C. suis*. With respect to the *E. bieneusi* genotypes, the EbpC, EbpA and PigEBITS5 subtypes were detected in pigs of all age categories; the HIJ-IV, CHG19 and H subtypes were detected only at weaning; the Henan-IV subtype was detected at weaning and finishing; the CHS5 subtype was detected at weaning and in sows; and the BEB6 and O subtypes were detected only in sows. Similarly, *Blastocystis* subtypes ST1, ST3 and ST5 were found in all age categories of pigs, whereas suckling piglets had only subtype ST5.

**Table 4:** Infections and genotypes of intestinal parasites in pigs

Factors	Number of pig fecal samples	Total number of positives	*Cryptosporidium* spp.		*E. bieneusi*		*Blastocystis* sp.	
			Number of positives	Species (*n*)	Number of positives	Genotypes (*n*)	Number of positives	Subtypes (*n*)
*Location*								
Henan	460	261	13	*C. scrofarum* (9), *C. suis* (4)	59	EbpC (38), EbpA (9), Henan-IV (4), CHG19 (3), PigEBITS5 (3), CHS5 (1), H (1)	189	ST1 (4), ST3 (2), ST5 (183)
Shaanxi	942	271	18	*C. scrofarum* (3), *C. suis* (15)	74	EbpC (34), EbpA (32), HIJ-IV (4), O (3), BEB6 (1)	179	ST1 (13), ST3 (14), ST5 (152)
Shandong	69	52	0	*-*	27	EbpC (12), EbpA (7), PigEBITS5 (4), CHS5 (2), CHG19 (2)	25	ST3 (3), ST5 (22)
Guangdong	108	90	2	*C. suis* (2)	21	PigEBITS5 (5), EbpC (13), EbpA (3)	67	ST1 (2), ST3 (3), ST5 (62)
Jiangsu	108	104	8	*C. scrofarum* (8)	45	EbpC (44), CHS5 (1)	51	ST1 (2), ST3 (2), ST5 (47)
Jiangxi	107	42	0	*-*	0	-	42	ST5 (42)
*Pig developmental stage*								
Suckling piglets (< 21 days)	248	58	0.4% (1/248)	*C. scrofarum* (1)	1.6% (4/248)	EbpC (2), EbpA (1), PigEBITS5 (1)	21.4% (53/248)	ST5 (53)
Weaning pigs (1–2 months)	444	319	6.5% (29/444)	*C. suis* (19), *C. scrofarum* (10)	24.8% (110/444)	EbpC (75), EbpA (15), PigEBITS5 (6), HIJ-IV (4), CHS5 (1), H (1), Henan-IV (3), CHG19 (5)	40.5% (180/444)	ST1 (4), ST3 (4), ST5 (172)
Finishing pigs (3–6 months)	670	193	0.9% (6/670)	*C. scrofarum* (5), *C. suis* (1)	6.5% (44/670)	EbpC (34), EbpA (7), PigEBITS5 (2), Henan-IV (1)	21.3% (143/670)	ST1 (2), ST3 (10), ST5(131)
Sows (> 6 months)	432	250	1.2% (5/432)	*C. scrofarum* (4), *C. suis* (1)	15.7% (68/432)	EbpC (30), EbpA (28), PigEBITS5 (3), CHS5(3), BEB6 (1), O (3)	41.0% (177/432)	ST1 (15), ST3 (10), ST5(152)
*Breeding scale*								
Large-scale	833	275	**16**		**79**		**180**	
Xianyang	200	20	0	–	1.5% (3/200)	EbpA (3)	8.5% (17/200)	ST5 (17)
Hanzhong	30	13	0	–	0	–	43.3% (13/30)	ST5 (13)
Xuchang	100	28	2.0% (2/100)	*C. scrofarum* (1), *C. suis* (1)	2.0% (2/100)	EbpC (2)	24.0% (24/100)	ST1 (1), ST5 (23)
Hebi	26	8	0	–	0	–	30.8% (8/26)	ST5 (8)
Xinyu	107	42	0	–	0	–	39.3% (42/107)	ST5 (42)
Xuzhou	108	104	7.4% (8/108)	*C. scrofarum (8)*	41.7% (45/108)	EbpC (44), CHS5 (1)	47.2% (51/108)	ST1 (2), ST3 (2), ST5 (47)
Weinan1	262	60	(3.0%) 6/262	*C. suis* (4), *C. scrofarum* (2)	(11.06%) 29/262	EbpC (15), EbpA (14)	(9.5%) 25/262	ST1 (3), ST3 (2), ST5 (20)
Small-scale	961	545	*25*		147		373	
Baoji	250	99	0.8% (2/250)	*C. scrofarum* (1), *C. suis* (1)	1.2% (3/250)	EbpC (3)	37.6% (94/250)	ST1 (6), ST3 (6), ST5 (82)
Linyi	29	20	0	–	31.0% (9/29)	EbpA (5), PigEBITS5 (4)	38.0% (11/29)	ST5 (11)
Heze	40	32	0	–	45.0% (18/40)	CHS5 (2), EbpA (2), EbpC (12), CHG19 (2)	35.0% (14/40)	ST3 (3), ST5 (11)
Zhoukou	87	58	8.0% (7/87)	*C. scrofarum* (7)	9.2% (8/87)	EbpA (6), EbpC (2)	49.4% (43/87)	ST1 (3), ST3 (1), ST5 (39)
Kaifeng	76	61	0	–	26.3% (20/76)	CHS5 (1), H (1), CHG19 (1), EbpA (2), Henan-IV (3), EbpC (12)	53.9% (41/76)	ST5 (41)
Xinyang	129	74	0	–	10.1% (13/129)	EbpC (13)	47.3% (61/129)	ST5 (61)
Nanyang	12	16	25.0% (3/12)	*C. suis* (3)	91.7% (11/12)	EbpC (6), CHG19 (2), PigEBITS5 (3)	16.7% (2/12)	ST5 (2)
Zhumadian	30	16	3.3% (1/30)	*C. scrofarum* (1)	16.7% (5/30)	EbpC (3), EbpA (1), Henan-IV (1)	33.3% (10/30)	ST3 (1), ST5 (9)
Heyuan	108	90	1.9% (2/108)	*C. suis* (2)	19.4% (21/108)	EbpC (13), EbpA (3), PigEBITS5 (5)	62.0% (67/108)	ST1 (2), ST3 (3), ST5 (62)
Weinan2	200	79	(5%) 10/200	*C. suis* (10)	(19.5%) 39/200	EbpC (17), HIJ-IV (4), O (3), EbpA (15)	(15.0%) 30/200	ST1 (4), ST3 (6), ST5 (20)
Total	1794	820	2.3% (41/1794)	*C. scrofarum* (20) *C. suis* (21)	12.6% (226/1794)	EbpC (141), EbpA (51), HIJ-IV (4), PigEBITS5 (12), Henan-IV (4), BEB6 (1), CHS5 (4), H (1), O (3), CHG19 (5)	30.8% (553/1794)	ST1 (21), ST3 (24), ST5 (508)

### Possible transmission of *Cryptosporidium *spp., *E. bieneusi* and *Blastocystis* sp. between pigs and NBFs

**Fig. 2:**
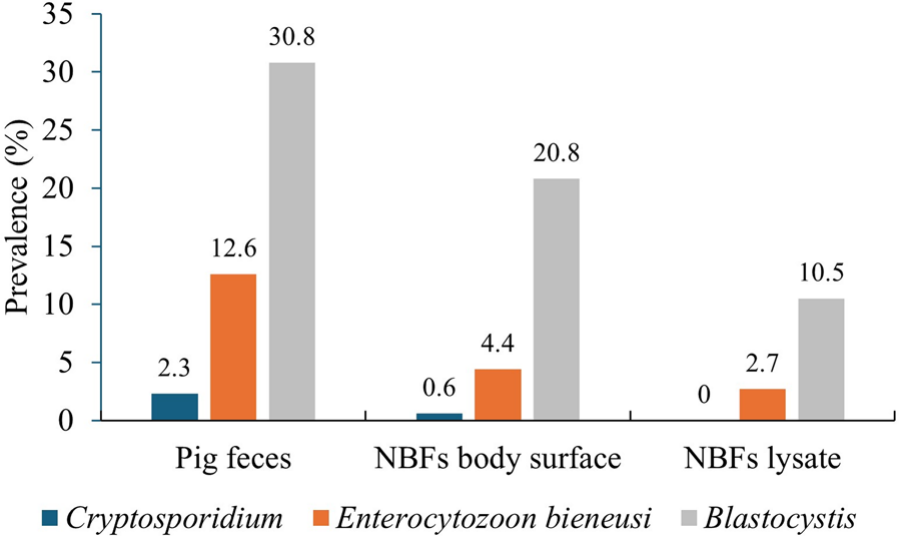
Proportion of *Cryptosporidium* spp., genotypes of *Enterocytozoon bieneusi* and subtypes of *Blastocystis* sp. detected on the body surface and in lysates of the sympatric non-biting flies (*NBFs*) collected from the pig farms

In the present study, the prevalence rates of *Cryptosporidium* spp*.*, *E. bieneusi,* and *Blastocystis* sp*.* were 2.3% (41/1794), 12.6% (226/1794) and 30.8% (553/1794) in pig feces; 0.6% (2/342), 4.4% (15/342) and 20.8% (71/342) on the body surface of NBFs; and 0% (0/342), 2.7% (9/342) and 10.5% (36/342) in NBF lysates as in vivo carriage (Fig. 2). One interesting observation in this study was that the co-prevalence of *Cryptosporidium* spp., genotypes of *E. bieneusi* and subtypes of *Blastocystis* sp. in pig feces and on the body surface and in lysates of the NBFs was found to be statistically significant (Chi-squared test,* p* < 0.05), suggesting that NBFs could play crucial roles in the distribution of these three protozoan parasites in pigs. The difference in the infection prevalence rates of the three parasites (*Cryptosporidium* spp., *E. bieneusi* and *Blastocystis* sp.) in pigs and on the body surface and in lysates of NBFs were compared using the Chi-squared test (*p* value < 0.05 was considered to be significant). The results showed a significant difference in the prevalence of these parasites in these groups of samples.

## Discussion

Non-biting flies naturally dwell in areas of filth, including pig farms, for feeding and breeding. Parasite cysts/oocysts readily adhere to the external body parts of NBFs, such as the proboscis, appendages, wings and bristles, while the flies are rest and moving around animal farms [1]. These flies not only facilitate the spread of parasites from one place to another but also lead to the contamination of food and water for animal consumption [15]. In the present study, morphological and molecular analyses revealed that 3420 flies (divided into 342 sample pools) collected from pig farms in six provinces in China were NBFs, with *M. domestica* being the dominant fly species. NBFs were found to carry *E. bieneusi* and *Blastocystis* sp*.* both on their body surface and in lysates, while *Cryptosporidium* spp*.* were detected only on the body surface. Our results confirmed that *M. domestica* from the same pig farms could use pig farms as abodes and contribute to the dissemination of *Cryptosporidium* spp., *E. bieneusi* and *Blastocystis* sp*.* In this context, we therefore suggest that different fly species can carry different species or genotypes of these intestinal protozoan parasites.

Different fly species can carry multiple parasites, and even a single fly species can serve as a vector for more than two parasite species [1]. NBFs can contaminate water, fruits, vegetables and animal feed mainly through surface contact and through their intestinal excretions [26]. Previous studies have shown that after the infection cycle, *Cryptosporidium* spp. infection by *M. domestica* can last up to 3 weeks, confirming the potential risk of mechanical transmission by *M. domestica* [27]. It has also been reported that the sequences of the *SSU* rRNA gene and *gp*60 locus of *C. parvum* from NBFs are 100% homologous to those of *C. parvum* from humans, suggesting that NBFs may be potential vectors of *C. parvum* [20].

NBFs are active mechanical vectors for the transmission of protozoan parasites [15, 28, 29]. In the present study, we identified *E. bieneusi* genotype EbpA from *M. domestica*, which is the first report of this genotype in this fly. Genotypes EbpA and EbpC both belong to Group 1 and are highly zoonotic. Although *E. bieneusi* genotype BEB6 belongs to Group 2, its detection in Swedish lambs [30], sheep in Inner Mongolia [31], red deer and Siberian roe deer [32], rodents, flies and cattle [21] and laboratory rodents [33] suggests zoonotic potential. EbpC is a genotype isolated only from NBFs associated with a wide range of infections, has been found in many mammals, including humans, and is classified as a Group 1 zoonotic [10]. The *Cryptosporidium* species identified in this study, *C. suis* and *C. scrofarum*, which were previously reported in humans, may pose a zoonotic risk. Screening for cryptosporidiosis in rural Madagascar identified cases of *C. suis* infection, suggesting the this species is highly likely to infect humans through contact with pigs or vector transmission [34]. The EbpC genotype of *E. bieneusi* has a wide host range and has been reported in humans and other animals [10].

**Fig. 3:**
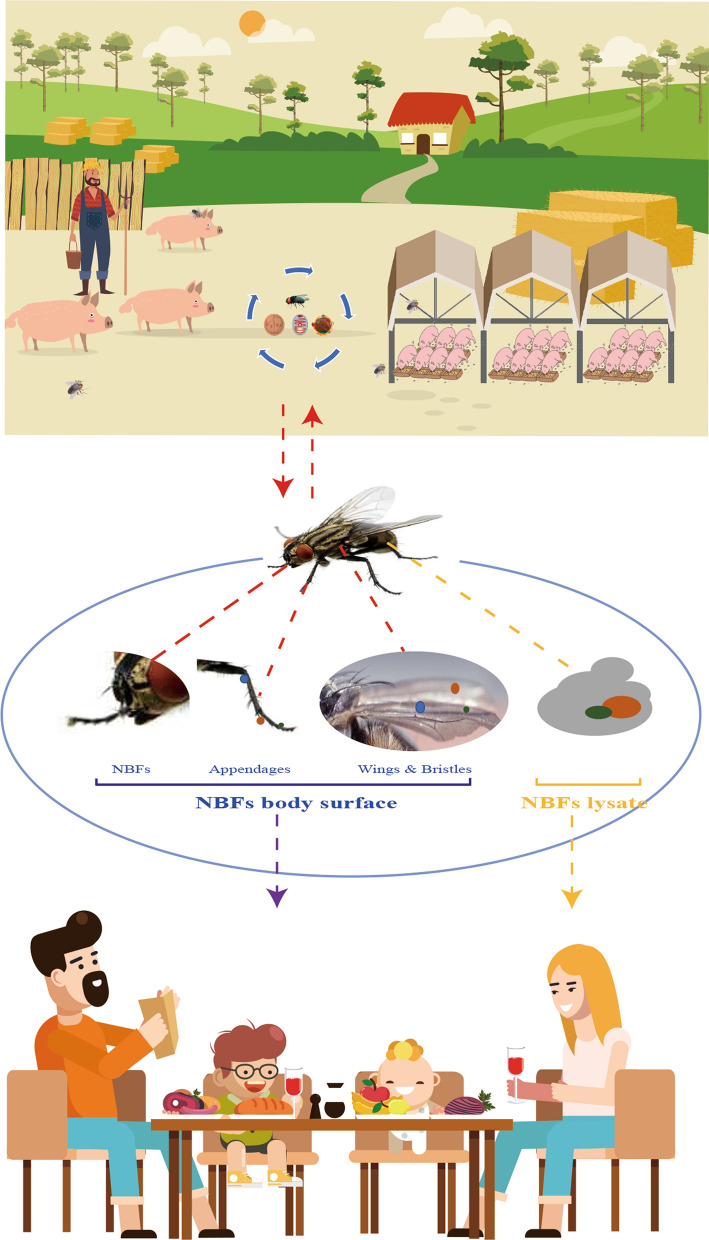
Illustration of possible transmission dynamics of zoonotic intestinal parasites by non-biting flies (*NBFs*)

In the present study, *Cryptosporidium* species *C. suis* and *C. scrofarum* were both identified in pig feces and on the body surface of the NBFs, which suggests possible transmission by NBFs as mechanical carriers (Fig. 3). Comparative genotype analysis revealed the co-occurrence of the *E. bieneusi* genotypes EbpC and EbpA and the *Blastocystis* species genotypes ST5 and ST3 in pig feces and on the body surface and in lysates of the NBFs, indicating a potential vector. Likewise, the *Blastocystis* species subtype ST1 was identified in pig feces and in lysates of NBFs, thus suggesting that the NBFs might deserve vectorial capacity in transmitting this parasite as well. Notably, certain proportions of the *E. bieneusi* genotypes EbpC (*n* = 5) and EbpA (*n* = 1) and the *Blastocystis* species genotypes ST3 (*n* = 9) and ST5 (*n* = 8) were identified both on the body surface and in lysates of NBFs. A critical consideration of this scenario implies that the sympatric NBFs deserve to be classified as potential vectors for zoonotic protozoan parasites on pig farms in China, as depicted in this study (Fig. 3). However, the exact transmission dynamics of these species/genotypes between NBFs and pigs on farms need to be confirmed by further detailed investigations.

A survey of hospitalized children in Shanghai revealed that EbpC was the predominant genotype associated with high transmission risk [35]. In addition, when the occurrence of *E. bieneusi* in river water contaminated by dead pigs in Shanghai was investigated, EbpC was found to be the most common genotype and to have a high potential to cause health threats to humans [36]. Although the same EbpA genotype was not identified in NBFs or pigs in the same locality in the present study, this genotype may represent a threat to swine health owing to the behavioral characteristics of NBFs. The absence of certain parasite genotypes in NBFs, especially on their body surface, might be attributed to the grooming behavior of the flies, as this behavior could remove attached parasites from their bodies [37]. Moreover, we suggest that the feeding habits of different species of flies and the different climates of the study region could be another factor for the disparity in the occurrence of same genotype of parasites in pigs and NBFs. The most common subtype of *Blastocystis* species, ST1, is highly zoonotic, whereas ST3 is a subtype of human infection reported in many animal hosts and humans [38]. Subtype ST5 has been detected in pigs and farm workers, indicating that this subtype has the potential to cause zoonosis [39]. The zoonotic risk of *Blastocystis* sp*.* transmission in pigs was demonstrated in a study in Queensland, Australia, where zoonotic subtype ST5 was detected in pigs and pig farm workers [36]. Subtype ST5 has also been identified in humans and pigs in rural areas of Jiangxi owing to the frequent contact between humans and pigs and mutual transmission, thus indicating its potential for zoonotic transmission [38].

 In summary, NBFs are mechanical vectors for various parasites, most of which are zoonotic, since NBFs may travel as vectors from humans to humans and from humans to animals, and vice versa. Acquiring data on monthly and seasonal variations in relation to the prevalence of parasites transmitted by NBFs would be an important focus of research. However, whether the extent to which these NBFs are vectors of the aforementioned parasites remains one of the important factors to be thoroughly investigated in the future studies. Regular inspection of pig farms for the presence of these NBFs and implementation of control measures are crucial aspects in mitigating the spread of zoonotic parasites among pigs, possibly to humans, and for standardizing the biosecurity with regard to One Health measures.

## Conclusions

Non-biting flies were found to carry *E. bieneusi* and *Blastocystis* sp*.* both on their body surface and in lysates, whereas *Cryptosporidium* spp. were detected only on the body surface. Notably, *M. domestica*, present at all pig farms as the dominant NBF, was found to serve as a potential vector for all the detected protozoan parasites. Furthermore, *C. suis*, *C. scrofarum*, *E. bieneusi* genotype EbpC and *Blastocystis* sp*.* subtypes ST1, ST5 and ST3 were identified in both of these vector NBFs and in host pigs on the same pig farms and all are zoonotic. Our results indicate that NBFs play a possible role as vectors in disseminating zoonotic *Cryptosporidium* spp., *E. bieneusi* and *Blastocystis* sp*.* between pigs. This finding highlights the importance of stringent control of NBFs on pig farms as a crucial biosecurity measure for protecting susceptible hosts.

## Supplementary Information

Additional file 1: Table S1. Sociodemographic characteristics of the study population of non-biting flies (NBFs) and pigs

Additional file 2: Table S2. Primers used to amplify cytochrome* c* oxidase I (CO1) gene of NBFs and three intestinal protozoa (*Cryptosporidium* spp., *Enterocytozoon bieneusi* and *Blastocystis* sp.)

## Data Availability

No datasets were generated or analysed during the current study.

## Abbreviations

COI: Cytochrome* c* oxidase 1
ITS: Internally transcribed spacer
NBFs: Non-biting flies
*SSU* rRNA: Small subunit ribosomal ribonucleic acid gene
