# Supplementary material for: Sympatric non-biting flies serve as potential vectors of zoonotic protozoan parasites on pig farms in China
Source: Parasit Vectors. 2025 Feb 18;18:59. doi: 10.1186/s13071-025-06686-2 (PMC11837656; doi:10.1186/s13071-025-06686-2)
Supplement: Supplementary file 2 — Additional file 2: Table S2. Primers used to amplify cytochrome c oxidase I (CO1) gene of NBFs and three intestinal protozoa (Cryptosporidium spp., Enterocytozoon bieneusi and Blastocystis sp.) [file 13071_2025_6686_MOESM2_ESM.docx]

**Table S2. Primers used to amplify cytochrome C oxidase I (COl) gene of NBFs and three intestinal protozoa (*Cryptosporidium*** **spp., *Enterocytozoon bieneusi* and *Blastocystis* sp.)**

| **Target** | **Primers (5’-3’)** | **Annealing temperature (****°C)** | **Fragment size (bp)** |
| --- | --- | --- | --- |
| COI | F: GGTCAACAAATCATAAAGATATTGG | 50 °C | 710 bp |
|  | R: TAAACTTCAGGGTGACCAAAAAATCA |  |  |
| *Cryptosporidium* spp.  18S rRNA | SSU-F2: TTCTAGAGCTAATACATGCG | 55 °C | 830 bp |
|  | SSU-R2: CCCATTTCCTTCGAAACAGGA |  |  |
|  | SSU-F3: GGAAGGGTTGTATTTATTAGATAAAG | 55 °C |  |
|  | SSU-R4: CTCATAAGG TGCTGAAGGAGTA |  |  |
| *Enterocytozoon bieneusi*  ITS | EBITS3: GATGGTCATAGGGATGAAGAGCTT | 57 °C | 392 bp |
|  | EBITS4: TATGCTTAAGTCCAGGGAG |  |  |
|  | EBITS1: AGGGATGAAGAGCTTCGGCTCTG | 55 °C |  |
|  | EBITS2.4: AGTGATCCTGTATTAGGGATATT |  |  |
| *Blastocystis* sp. SSU rRNA | RD5: ATCTGGTTGATCCTGCCAGT | 55 °C | 600 bp |
|  | BhRDr: GAGCTTTTTAACTGCAACAACG |  |  |
